# A Call for Biomonitoring Systems in Latin America and the Caribbean: Considerations for Potentially Toxic Metals/Metalloids

**DOI:** 10.5334/aogh.3637

**Published:** 2022-09-14

**Authors:** Marcela Tamayo-Ortiz, Horacio Riojas-Rodríguez, Martha M. Téllez-Rojo, Ana Boischio, Nelly Mañay, José Antonio Menezes-Filho, Elena I. Queirolo, Sandra Cortés, Katarzyna Kordas

**Affiliations:** 1 Occupational Health Research Unit, Instituto Mexicano del Seguro Social, Mexico City, Mexico; 2 Research Center for Population Health, National Institute of Public Health, Cuernavaca, Mexico; 3 Research Center for Health and Nutrition, National Institute of Public Health, Cuernavaca, Mexico; 4 Cambio climático y determinantes ambientales de la salud, Organización Panamericana de Salud, Washington, DC, USA; 5 Toxicology area, Faculty of Chemistry, Universidad de la República UDELAR, Montevideo, Uruguay; 6 Graduate Program in Pharmacy, College of Pharmacy, Federal University of Bahia, Brazil; 7 Department of Neuroscience and learning, Catholic University of Uruguay, Uruguay; 8 School of Medicine, Pontificia Universidad Católica de Chile, Advanced Center for Chronic Diseases (ACCDIS), Centro de Desarrollo Urbano Sustentable (CEDEUS), Chile; 9 Department of Epidemiology and Environmental Health, University at Buffalo, USA

**Keywords:** biomonitoring, Latin America, Metals

## Abstract

The Latin America and the Caribbean (LAC) region makes up 8.4% of the world’s population. Human biomonitoring (HBM) programs, which can shed light on population-level exposure to environmental contaminants such as toxic metals and thus, improve the health of the populations are inexistent in LAC countries. We call for the creation of HBM programs in the region and identify three viable design options for HBM at the individual level, through national surveys, newborn blood collection, and biobanks. We highlight some of challenges to the implementation of HBM programs, including financial and human resources, technical constrains (laboratory), sample, and data logistics. Finally, we provide the case studies of Brazil, Chile, Mexico, and Uruguay, to illustrate a range of responses to toxic metal exposure in non-occupational populations. Although different in many respects, the individual country responses share two commonalities: 1) academic centers drive the research to raise awareness of governmental entities; 2) reference levels are adapted from international norms rather than arising from the studied populations. Well-designed and sufficiently funded biomonitoring systems need to be established in each country of the LAC region. HBM programs are key to promoting human health by informing the public and contributing to policy processes that establish sustainable environmental controls and health prevention programs.

## Introduction

According to United Nations 2019 statistics, 651 million people live in Latin America and the Caribbean (LAC), making up 8.4% of the world’s population [1]. By mid-2050, that number is projected to increase to 758.9 million (7.7%). Chemical contaminants are important determinants of health and disease in LAC, where 79% of people live in urban centers, 37% inhabiting cities of 1 million or larger [2]. Unfortunately, the true magnitude and sources of contaminant exposure in LAC populations are not well understood [3]. Exposure to toxic metals and metalloids in LAC has received some attention in the academic literature, with lead (Pb) and mercury (Hg) being studied most frequently and additional reports focusing on cadmium (Cd), inorganic arsenic (iAs), manganese (Mn), particularly among children [4]. The epidemiological studies conducted on these topics have: 1) been driven mostly by academic or medical research or health centers, 2) focused on high-risk groups (children or pregnant women), specific geographic areas often with high levels of contamination, or occupational groups, 3) typically collected data on small numbers of individuals or at a single time point, and 4) depended on funding trends to determine their longevity and scope. We call on LAC country governments to develop and implement Human Biomonitoring (HBM) programs to generate comprehensive, consistent, and long-term evidence on environmental exposure to contaminants such as metals and metalloids, and to inform policies for the protection of human health.

Our definition of HBM programs is the “systematic and continuous or repetitive activity for collection of biological samples for analysis of concentrations of pollutants and its metabolites, with the objective to assess exposure, changes after specific interventions and standardized protocols that allow to compare the data observed over time and with reference levels and—if necessary—leading to corrective actions”[5]. With HBM, we focus on dose monitoring—the measurement of toxic chemicals or their metabolites in human body tissues, and on environmental exposures in the general population, as opposed to occupational groups. We selected metals and metalloids due to the observed deleterious effects related to the exposure in developed countries, but also for the environmental levels described in LAC, especially in countries with mining and industrial activities. We also distinguish HBM from the complementary activity of ambient monitoring, which is the measurement of chemicals in environmental samples, including air, water, soil and food [6]. HBM reflects the internal dose of chemicals taken up from any or all sources in the environment. HBM has been applied in different countries as a tool for exposure and risk assessment to chemicals in the environment, and the results contributed to advances in policies to protect susceptible populations such as children and pregnant women. This is the case of the high-income countries in North America and Europe or Asia that have included HBM for relevant contaminants, such as metals, but also pesticide metabolites, phthalates and persistent organic pollutants (POP) into their national health surveys [7].

In most cases, non-governmental organizations, including academia and health of individual countries and regional health organizations in LAC, recognize the importance of environmental factors to human health (e.g. PAHO Atlas of Children’s Health and the Environment) [8]. Yet, despite clear benefits, very limited resources exist for HBM programs at an individual country or regional level in LAC. Under the Stockholm Convention on POPs, a global survey of organic contaminants in human milk has been implemented, with the participation of Antigua and Barbuda, Chile, Mexico, Argentina, Colombia, Peru, Barbados, Ecuador, Uruguay, Brazil, Jamaica. Health is directly addressed in the Minamata convention on mercury adopted by the World Health Organization Assembly. All 20 countries in Latin America and most countries in the Caribbean have signed and ratified the convention (WHA 67.R11) [9] for the required actions, including HBM programs. These actions are a step in the right direction, but more is needed to protect human health.

To stimulate the conversation, our objective here is to consider potential design options, as well as openly discuss the challenges, for the development of HBMs for metals and metalloids in the LAC context. Furthermore, focusing primarily on toxic metals/metalloids, we include case studies from Brazil, Chile, Mexico, and Uruguay to illustrate the range of responses in the entire region to the need for accurate and timely information on population-level environmental exposures. Whereas we recognize the importance of exposure to numerous toxic or potentially toxic substances in the region, including fluoride [10,11], our aim was not to perform an exhaustive review of exposures but rather to focus on those that have received more attention with regard to HBM in LAC, hence our focus on metals/metalloids.

## Considerations for the Design of Biomonitoring Systems

Various options exist for the design of a HBM system in LAC that can be tailored to fit the available financial and human resources, and the infrastructure in place in each country. We briefly outline three program options, along with design elements that need to be considered, which we view as viable in LAC countries (Table 1). Detailed discussion of the preparation and organization of HBM studies is available elsewhere [5,12].

**Table 1:** Design options, considerations, and elements for Human Biomonitoring Programs in LAC countries.


DESIGN PLAN ELEMENTS	HUMAN BIOMONITORING PROGRAM – DESIGN OPTIONS AND CONSIDERATIONS		

	INDIVIDUAL/CENSUS (ALL INDIVIDUALS PARTICIPATE)	SURVEY (INTEGRATED INTO HEALTH EXAMINATION POPULATION SURVEYS)	BIOBANK (BLOOD BANKS, BREASTMILK BANKS, BLOOD DONORS)

	Pro: Comprehensive; can utilize existing health system infrastructures (ex., vaccination campaigns, well-child visits); allows QC in bio-sample collection and storage. Con: Very costly. Challenges for implementation.	Pro: Utilizes existing infrastructure; allows periodic monitoring, allows QC in bio-sample collection & storage. Con: requires knowledge of probabilistic sampling.	Pro: Utilizes existing infrastructure; allows continuous monitoring; ensures sufficient sample volume. Con: Convenience sample; possibly low age, sex- and regional representativeness; lower possibility of QC in bio-sample collection & storage.

Participating Institutions	Involvement of national-level research or regulatory institutions, along with central government funding ensures program success and sustainability for use of data and information.		

Target population	Can include the general, non-institutionalized population, be age-specific (neonates—via umbilical cord blood sampling or assays in neonatal blood spot; children) or focus on particularly vulnerable (ex., pregnant women) populations. Some programs over-sample for ethnic/racial groups or poverty, calculating survey weights to extrapolate information to total populations. Multi-stage sampling strategies are common to achieve nationally representative sample.		

Specific national context	Can cover all geographic areas of a country or be area-specific according to national priorities (ex., oversampling in regions of specific concern).		

Sampling frequency	Can be continuous/repeated annually or conducted occasionally (ex., every 5 years). We advocate for the former.		

Sample criteria and size	Based on variation in the biomarker measures, enough individuals should be randomly recruited to provide reliable area or national estimates, depending on the geographical coverage. ** does not apply to the Individual/Census		

Matrix selection	Should consider feasibility of collecting biological samples, cold chain maintenance, assurance of appropriate storage conditions, and generation of biomarkers that have established reference values.		

Biomarkers of exposure*	Can include a comprehensive suite of toxic metals or a core set of metals representing greatest concern for the population or highest possible health risk. We advocate for the inclusion of Pb, Mn, Hg, Cd, As. At a minimum, Pb should be assessed.		

Laboratory capability	IC-PMS techniques can detect over 20 major or trace metals/metalloids in blood, urine or hair in a single run and have low limits of detection, allowing for measurement of low-level exposures. IC-PMS requires a high level of initial investment and maintenance costs and trained personnel. Method validation is required prior to bio-sample analysis.		

Laboratory QC/QA	Quality control and assurance programs need to validate laboratory processes and verify results. Consistent laboratory operations are required to produce reliable and consistent results. Use of “round robin” tests ensures reproducible protocols among laboratories.		

Use & dissemination of data	Open sharing of de-identified or anonymized data (most preferable) promotes transparency, research, public education and policy formulation around environmental contaminants and health threats. Restricted-access data on geographical location of participants could contribute to addressing area-specific research questions.		


* Biomarker of exposure is defined as an exogenous substance or its metabolite or the product of an interaction between a xenobiotic agent and some target molecule or cell that is measured in a compartment within an organism [59].

## Challenges for Implementation

**Figure 1:**
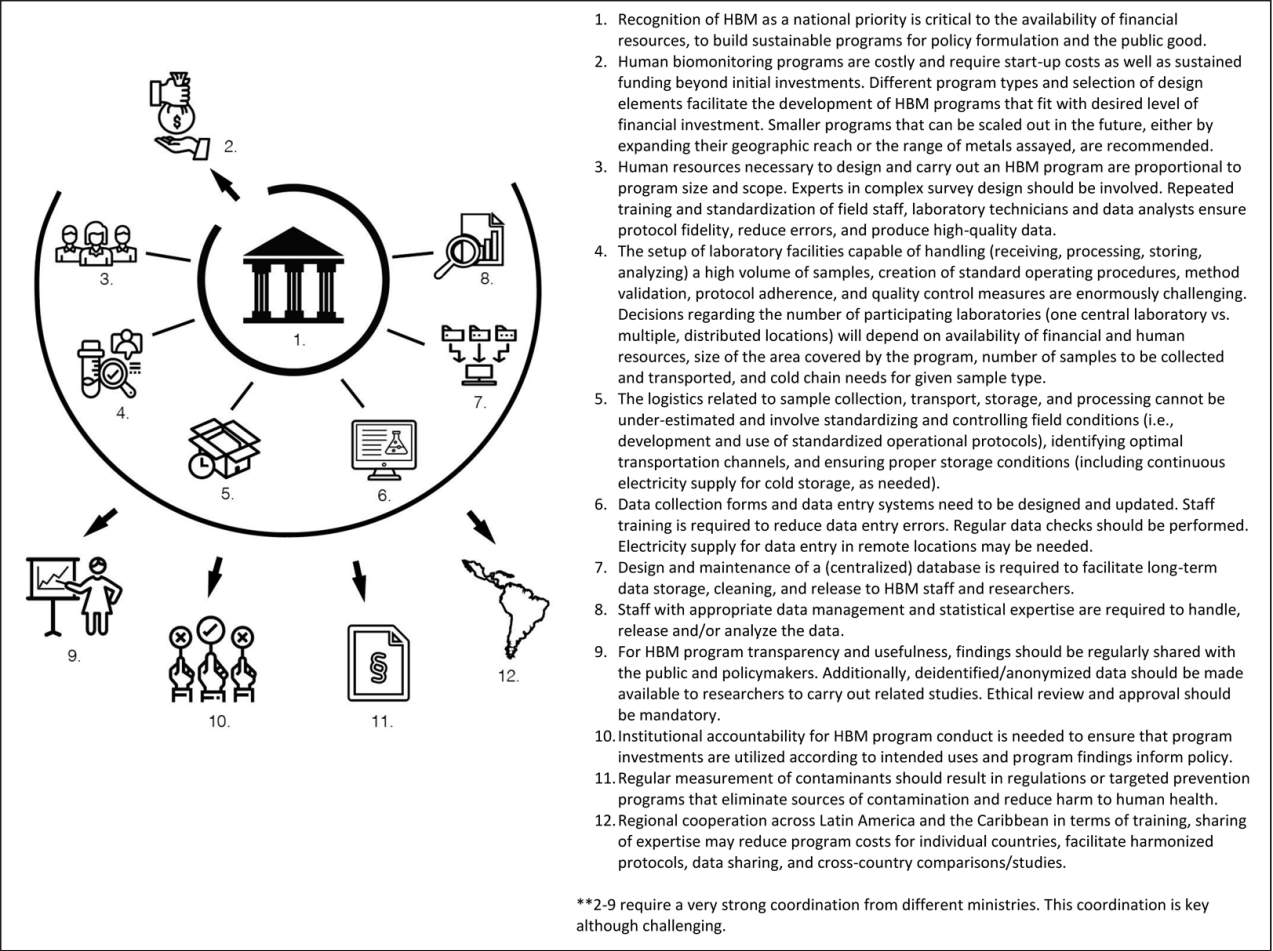
Key challenges to Human Biomonitoring Program implementation in LAC countries.

There are several challenges to developing a well-functioning HBM (Figure 1), starting with the recognition of HBM as a national priority in each LAC country. We also highlight how factors such as finances, human and physical resources, laboratory and well-trained teams, sample and data logistics represent additional challenges. Nevertheless, strong institutional support, sufficient financing, and integration of HBM into existing facilities are key to creating successful and sustainable programs. We also recognize that a region-wide program is not likely feasible; however, there are several actions that could enable regional cooperation.

## Case Studies

In consultation with academic colleagues living and/or working in LAC, we notice that there are no HBM programs, as we envision them, currently in place in the region. The examples below illustrate how information about toxic metal/metalloid exposure is currently generated and used in LAC more broadly.

### Brazil

Is a country of >200 million inhabitants and immense social inequalities, there are currently 23.3 million people living below the poverty line, which rose to 33% in the last four years [13]. Poverty is a strong social determinant of exposure to potentially toxic metals [14,15]. Vulnerable people live in polluted areas and close to industrial sites, solid waste deposits, highways, and regions without public sanitation. Brazil has a National Institute of Health and a broad unified health system (SUS) but lacks a national HBM program. Such a program would require considerable financial and logistical resources similar to the national vaccination program. Political will to assess the most disadvantaged fraction of the population is lacking. Most exposure assessment to date has been carried out by universities, state, or federal research institutes.

Lead exposure has received most attention. The city of Santo Amaro, Bahia, was contaminated by atmospheric emissions and solid waste dumping from a foreign primary smelting company for decades until its closure in the 1990s. Researchers reported intense occupational and environmental exposure and health effects on local inhabitants, especially children [16].

Artisanal and small-scale gold mining (ASGM) operations have been considered the most significant source of atmospheric mercury emissions, with estimates based on 2015, of around 37.7% of the global 2220 tons. In LAC including countries like Brazil, Bolivia, Colombia, Perú, Ecuador and Mexico, ASGM accounts for around 80% of the total 454 tons of atmospheric mercury emissions [17]. Despite the challenges for precise estimates of gold production by ASGM in Brazil, there are accounts that this sector includes more than 400 000 miners, considering both formal and informal sectors [18]. These workers, their families and nearby communities are likely exposed to metallic mercury due to amalgam burning and to methyl mercury through fish consumption. An HBM program should consider urine for metallic mercury and hair for methylmercury (emphasizing adequate wash of exogenous mercury along hair strands before analytical measurements given the degree of atmospheric mercury contamination likely found in these ASGM communities). Riverside populations living in tropical forests, have been considered the most critically exposed to methylmercury [19]. In the Amazon, links between hair methylmercury levels and cognitive, motor, and visual impairments [20,21] have been observed.

In the last 15 years, manganese exposure has been assessed in regions like Bahia, where a ferro-alloy plant has been releasing thousands of tons of Mn oxides into the atmosphere since the 1970s. Cognitive and behavioral effects were reported [22,23]. It has also been shown that in Rio Grande do Soul, the use of the fungicide Mancozeb in tobacco and vine crops contributes to community exposure to Mn [24].

### Chile

Arsenic exposure has been broadly studied between the years 1958 to 1970 in drinking water in Antofagasta, Northern Chile [25]. This long-term exposure to inorganic arsenic (iAs), at levels above 600 µg/L, has been associated with increased mortality, cardiac infarctions, and pulmonary, vesical, and renal cancer. After the 1970s, water treatment plants started their operations to reduce levels of inorganic arsenic in drinking water, without acute effects in population along the country. In the 1990s blood lead levels (BLL) in Chilean children dropped after leaded gasoline was phased out of use [26], with beneficial effects at national levels.

Other limited studies had been conducted in local communities, which allows identifying concentrations of metals in communities where pollutants were disposed of without any control. Public concerns were observed in Arica, North of Chile, by a waste disposal site containing heavy metals and cognitive performance in children exposed at the end of the 1990s [27]. The so called “Arica Case” represented a change in the public policies, by a law to ensure preventive measures in the affected area; from the year 2015, several clinical guidelines were developed considering expert opinion for the biological surveillance of the exposed population. Some mining communities have been exposed to metals for a long time, without any response from governmental agencies. Chañaral, located in the north of Chile is an emblematic case, where the population showed high levels of several metals [28].

In the 2016–17 National Health Survey, metal exposure was measured in a subsample of 3 547 adult volunteers from the whole country. Urinary iAs concentrations had a median of 12.04 µg/L (min-max 5.0 to 288.41 µg/L); BLL was 1.00 µg/dL (1.00–26.75 µg/dL), urinary mercury 2.00 (2.99–16.33 µg/L); urinary cadmium 1.00 (1.00–10.27 µg/L). At the national level, the prevalence of elevated exposure was highest for iAs (≥35 µg/L). This study was the first time that metals were measured in the National Health Survey, to have a preliminary baseline of exposure, with strong differences along the country. Despite the low levels detected for the metals evaluated, the next national health surveys are expected to repeat these measurements and explore relationships with health outcomes. Until now, we do not have specific HBM studies for pregnant women or children. Additional studies in Antofagasta showed that 8% of adults and 12% of children had elevated iAs. The other metals were below the risk levels defined by the health authority (10 µg/L for chromium, 10 µg/L for mercury, 2 µg/L for cadmium, 5 and 10 µg/dL for BLL in children and adults, respectively) [29]. Other biomonitoring studies will be carried-out in the near future to describe the exposure to metals in two areas located near industrial parks, in a collaborative effort from the Ministry of Health and the academia.

### Mexico

Between 1994 and 2005 Mexico collaborated with the Commission for Environmental Cooperation [CEC] in monitoring and training pilot programs with the aim of establishing a biomonitoring program. This collaboration resulted in publications comparing POPs and metals concentrations in blood between the U.S., Canada and Mexico [30], and POP concentrations in maternal milk across Mexico [31]. Despite these joint efforts and unlike its CEC partners, Mexico was unable to establish a biomonitoring system. The lack of political will, institutional capacity and investment in necessary laboratory infrastructure contributed to this situation.

Lead is another well known, systematically unmonitored exposure in Mexico. Evidence from epidemiological studies in specific populations documented the decreasing trend of BLLs as a result of introducing lead-free gasoline in 1990 [32]. This trend has plateaued, pointing at other current sources of lead exposure, where the use of low temperature lead-glazed ceramics (LTPbC), a Mexican cultural legacy, has been identified as the most important. A state-representative, hospital-based study of newborn cord BLLs documented the magnitude of the problem and promoted the first national-level study in the 2018 National Health and Nutrition Survey (NHNS) [33]. An estimated 17.4% children ages 1–4 years had BLL≥5µg/dL, approximately 1.4 million, with LTPbC as the main source of exposure. In November 2019 Mexico’s General Health Council (GHC) approved an initiative for a national chemical substances control policy, supporting the possibility of a biomonitoring program [34]. Leveraging the NHNS results and the unquestionable toxicity of lead, the GHC initiative has begun with lead regulation with the aim of expanding to other environmental toxicants in the future.

In Mexico, mercury is found in products that use it for their functioning, medical equipment, and mining extraction for domestic consumption and export. Vulnerable groups include ASGM in some areas of the country who use mercury to obtain gold and workers in mine extraction mainly in the state of Querétaro. Following the commitments of the Minamata agreement, Mexico is progressively reducing the use of mercury in medical devices and other products until its elimination. There is also a commitment to close the mines before 2030. The decree with Mexico’s commitments entered into force in 2017 with periodic progress evaluations [35].

### Uruguay

Lead poisoning became a recognized public health problem in Uruguay following a discovery in 2001 that children in a former industrial neighborhood (La Teja) of Montevideo were exposed to lead [36]. It was due to societal pressure that Uruguay began to acknowledge and face a problem that up to this point had been unrecognized [37]. That same year, a group of legislators in the nation’s House of Representatives introduced legislation that proposed the development of national studies to determine the prevalence of lead exposure in the pediatric population in Uruguay [38]. In 2008, based on the determination of health authorities and academic experts that the problem of lead had been controlled, the legislation was tabled without passing on to the Senate [39].

To our knowledge, there is no systematic biomonitoring of toxic metals in Uruguayan populations not exposed occupationally. With respect to lead, there are several sources of exposure in Uruguay including water, paint, metallurgical and other industries as well as polluted areas, industrial waste sites and informal metal recycling activities [40].

Since 2009 the Health Ministry has a protocol for the management and follow up of children according to their BLL (MSP 123/2009) [41]. This protocol had been available on the Ministry of Public Health webpage [42], but is no longer accessible. Currently, the majority of studies evaluating children’s exposure to lead and other toxic metals are being carried out by Uruguayan academic centers and their collaborators [43,44].

## Discussion

A *Lancet* Commission report acknowledged that environmental pollution—air, water, soil, and chemical contamination—is an important contributor to disease and premature death, accounting for 16% of all deaths worldwide in 2015 alone [45]. WHO estimates that globally, around 1.6 million lives and 45 million disability-adjusted life-years were lost in 2016 due to selected chemicals, acting as risk factors associated with prevalent chronic diseases. Pollution also accounted for 940 000 deaths among children in 2016 alone, two-thirds of them under five years of age [46]. In LAC, children experience several environmental exposures including lead, and the documented exposure levels are detrimental to children’s neurodevelopment [47,48]. Lifelong trajectories of disease, from early origins to later-life manifestations, mean that exposure to pollution in childhood can affect human health along the entire continuum of life [49,50]. Unsurprisingly, pollution contributes to reductions in productivity and gross domestic product, as well as higher healthcare expenditures [45]. Exposure to many contaminants in LAC, particularly toxic metals, and POPs, can be attributed to anthropogenic sources, including heavy traffic and industrial production that are concentrated in urban centers, and to blurred lines between industrial and residential zones, or between urban and rural areas.

Most of the evidence on population exposures to toxic metals in LAC countries appears in academic publications and is often motivated by interest from local communities demanding attention from governments or academia. In all cases, reference limits for different toxicants have been established according to population norms from other countries or from occupational limits. HBM programs would allow individual countries to determine where their populations fall vis-à-vis the published norms and establish their own reference values. Furthermore, HBM provides important societal benefits, including the identification of temporal and geographic trends in exposure to contaminants, of high-risk sub-populations, of age or sex-related differences in toxicant metabolism, detection of exposure to emerging chemicals, and the evaluation of the effectiveness of public health interventions [5]. Together with epidemiological surveillance and specific studies in high risk populations, HBM can help elucidate early health effects of environmental exposures at a population level [6]. Finally, HBM has policy relevance at country and regional level (for suggesting priorities and measures of control or intervention). Clear examples of well-functioning HBM programs exist in the United States [51], Canada [52], Korea [53], and Europan nations (ex., Germany [54], Belgium [55], France [56], Czech Republic [57]). Ongoing efforts are leading to the creation of a unified HBM program across the European Union [58].

It is important to recognize that HBM programs are costly to start and maintain, and many challenges exist to their implementation. In this line of thought, starting with metals and metalloids that have a widely documented toxicity can be important; moreover, productivity and reduction of health care cost can greatly compensate an investment in HBM programs. We offer a frank view of these challenges in the belief that once they are identified, they can be analyzed in the context of each LAC country, and systematically addressed. Furthermore, once the political will to implement regular population-based monitoring of chemical exposures is in place, several HBM program design options exist that can be adapted to fit the existing resources. The three options we outlined offer a starting point for program design by individual countries or regionally. Notably, with time and documented success, programs could be scaled to include larger portion of the population and greater number of contaminants.

## 6. Conclusion

To gain an accurate and timely picture of environmental exposures in the general population of LAC populations, well-designed and sufficiently funded HBM programs need to be established. These programs are key to promoting human health by raising public awareness around contamination, stimulating research, and informing regulatory and policy processes.
